# Biomechanical Evaluation of a Novel Apatite-Wollastonite Ceramic Cage Design for Lumbar Interbody Fusion: A Finite Element Model Study

**DOI:** 10.1155/2018/4152543

**Published:** 2018-01-18

**Authors:** Celal Bozkurt, Alpaslan Şenköylü, Erdem Aktaş, Baran Sarıkaya, Serkan Sipahioğlu, Rıza Gürbüz, Muharrem Timuçin

**Affiliations:** ^1^Department of Orthopedics and Traumatology, Harran University School of Medicine, Osmanbey Kampusu, Mardin Yolu 20. Km, Haliliye, 63190 Şanlıurfa, Turkey; ^2^Department of Orthopaedics and Traumatology, Gazi University School of Medicine, Emniyet Mh Mevlana Bulvarı, Beşevler, 06500 Ankara, Turkey; ^3^Saglık Bakanlıgı Ankara Eğitim ve Arastırma Hastanesi, Sukriye Mh. Ulucanlar Cd. No. 89, Altındag, 06340 Ankara, Turkey; ^4^Department of Metallurgical and Material Engineering, Middle East Technical University, Üniversiteler Eskisehir Yolu No. 1, Cankaya, 06800 Ankara, Turkey

## Abstract

**Objectives:**

Cage design and material properties play a crucial role in the long-term results, since interbody fusions using intervertebral cages have become one of the basic procedures in spinal surgery. Our aim is to design a novel Apatite-Wollastonite interbody fusion cage and evaluate its biomechanical behavior in silico in a segmental spinal model.

**Materials and Methods:**

Mechanical properties for the Apatite-Wollastonite bioceramic cages were obtained by fitting finite element results to the experimental compression behavior of a cage prototype. The prototype was made from hydroxyapatite, pseudowollastonite, and frit by sintering. The elastic modulus of the material was found to be 32 GPa. Three intact lumbar vertebral segments were modelled with the ANSYS 12.0.1 software and this model was modified to simulate a Posterior Lumbar Interbody Fusion. Four cage designs in different geometries were analyzed in silico under axial loading, flexion, extension, and lateral bending.

**Results:**

The K2 design had the best overall biomechanical performance for the loads considered. Maximum cage stress recorded was 36.7 MPa in compression after a flexion load, which was within the biomechanical limits of the cage.

**Conclusion:**

Biomechanical analyses suggest that K2 bioceramic cage is an optimal design and reveals essential material properties for a stable interbody fusion.

## 1. Introduction

Interbody fusions using intervertebral cages have become a basic procedure within spinal surgery to treat degenerative disc disease and spondylolisthesis. Interbody fusion cages restore disc height and also provide stability for functional spinal units [1]. Cage design and material both play crucial roles in long-term results. Many types of cages are available in the market, made from a variety of materials, including titanium, carbon fibre, and polyetheretherketone (PEEK) [2]. A few studies have reported that although the biomaterials that are used for the production of cages provide sufficient mechanical support, there is no direct osteointegration between the host bone and these materials; instead, a fibrous tissue forms between the cages and the host bone [3].

Apatite-Wollastonite (A/W) bioceramic composite is a bioactive and compatible material that is used for hard-tissue repair. Its degradation rate is faster than that of hydroxyapatite (HA), which shows reduced solubility due to its chemical stability. The A/W bioceramic composite induces bone growth three times faster than HA [4–6]. Recent studies have concluded that direct osteointegration occurs between the host bone and the A/W bioceramic composite material and that this connection increases with time [7]. In addition to this action, the ceramic-like composite also increases mineral concentration in adjacent tissues. Over time, as this bioceramic composite degrades and is replaced by bone, its mechanical support function gradually decreases [8].

Many clinical conditions of the spine can be simulated using the finite element method (FEM) to provide biomechanical insight into the construct and repair of the spine [9]. The advantage of FEM is that it can estimate the stresses within spinal ligaments, intervertebral discs, and other tissues related to the spine, which may be both technically difficult and time-consuming to do experimentally. Another benefit is the detailed motion analyses that can be utilized by this method. As a result, FEM investigators can ascertain the relationships and mechanisms between the implant and the related spinal segments. Because many different surgical procedures and treatment options can be simulated with FEM, only the most suitable implants need to be tested before the production process begins [10].

The research questions that this study seeks to answer are as follows: (1) Can we produce an A/W bioceramic composite interbody fusion cage that is sufficiently robust to withstand various physiologic loading levels? (2) Do different cage geometries have a distinct effect on mechanical behavior (i.e., stress and strain)?

## 2. Materials and Methods

### 2.1. Production of A/W Bioceramic Composite Cage

Our ceramic samples contain hydroxyapatite (47.5%), pseudowollastonite (PW, 47.5%), and frit (5%; Sigma Aldrich Corp., USA). The HA powder was produced by the method developed by Akao et al. [12]. The wollastonite component of the ceramics was prepared via a thermal synthesis process. The product, identified by X-ray diffraction (XRD) as pseudowollastonite (*α*-CaSiO3), was milled to fine powder. The frit beads (Na2O-CaO-Al2O3-SiO2) were crushed and ground to a fineness below 3 *μ*m in size; prototype cages were then produced from this powder. The dimension of the cage produced was 15.5 mm × 15.5 mm × 10.2 mm. The final geometry of the prototype cages was obtained via a Challenger 2412 Microcut machine.

### 2.2. Unprocessed Prototype Cage Biomechanical Test

**Figure 1:**
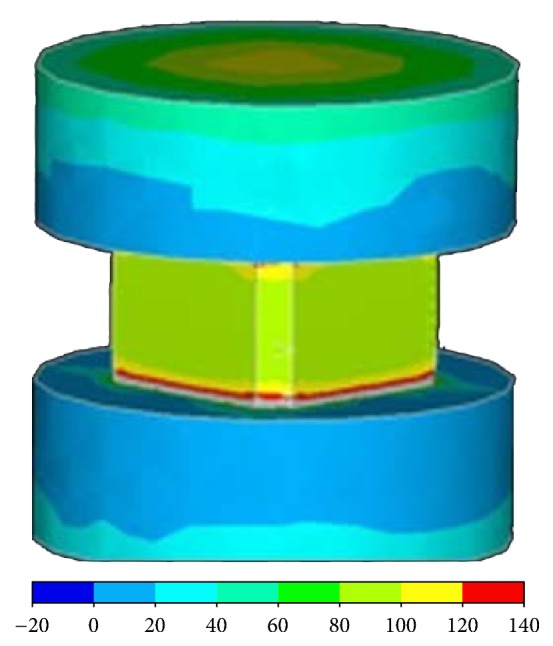
Two metal platens and the prototype cage modelled to perform a compression test with an Instron testing machine and video extensiometry.

A compression test was performed with an Instron testing machine and video extensiometry (Instron 5582 floor-mounted material testing system, Instron Products, USA) and an elastic modulus of the material was found; this test was then simulated with FEM. Two metal platens and the prototype cage were modelled for this purpose. The elastic modulus of the metal platens measured 200,000 N/mm^2^, with a Poisson ratio of 0.3. A 25,000 Newton (25 kN) axial load was applied to the system (Figure 1).

### 2.3. FEM of the Intact Lumbar Vertebrae

The computerised tomography (CT), magnetic resonance imaging (MRI), and colour sections of a male cadaver which may be found in the Visible Human Project (National Library of Medicine, National Institutes of Health, USA) database were converted to a surface model by the use of 3D-Doctor 3.5.050106 software (Able Software, USA). The surface model was then converted to a solid model using Autodesk AutoCAD 2005 (Autodesk, Inc., USA).

The solid model was then transferred to Ansys 12.0.1 (Ansys Inc., USA). The model included vertebral bones, intervertebral disks, end plates, posterior elements, and ligaments (supraspinous, interspinous, transverse, posterior longitudinal, anterior longitudinal, and flavum). The material properties were assumed to be homogenous and isotropic; the mechanical data were gathered from the literature [11] (Table 1).

The ligaments were modelled as two-point elements that were resistant to all but distraction. Their anatomic locations and cross-sectional areas were in concordance with the literature [11].

The surface-to-surface frictional areas between biological tissues were simulated assuming a friction coefficient of 0.1; the distance between the facet joint surfaces was assumed to be 0.5 mm. The connections of the rods to the pedicle screws were assumed to be rigid. The diameters of the rods and screws (made of titanium) were assumed to be 6 mm.

The intact model was comprised of 77,282 nodes and 48,172 elements. The instrumented models' node and element numbers changed according to the geometry of the cages. The instrumented models were comprised of 102,000 nodes and 59,000 elements on average.

### 2.4. FEM of the Lumbar Interbody Fusion Model

**Figure 2:**
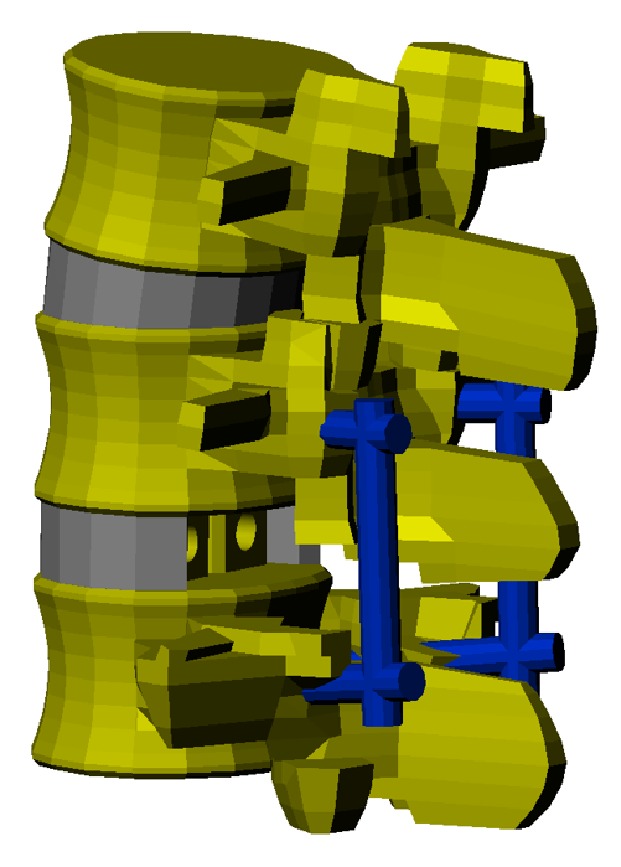
Following bilateral facetectomy and partial discectomy performed in silico in the L4-L5 motion segment to simulate posterolateral interbody fusion (PLIF), cages and a posterior instrumentation system are implanted.

Bilateral facetectomy and partial discectomy were performed in the L4-L5 motion segment to simulate posterolateral interbody fusion (PLIF); cages and a posterior instrumentation system were then implanted. Four pedicle screws (*r* = 6 mm) were implanted into the L4 and L5 lumbar vertebrae. Screws were connected with two rods (*r* = 6 mm). The screws and rods were modelled with beam elements. Two cages were placed into the intervertebral space. A postoperative bone-cage interface was then modelled using surface-to-surface contact elements. These contact elements were defined to transfer only compressive forces; they did not transfer distraction forces. The interface friction coefficient between implant and bone was assumed to be 0.8, in accordance with the literature (Figure 2).

**Figure 3:**
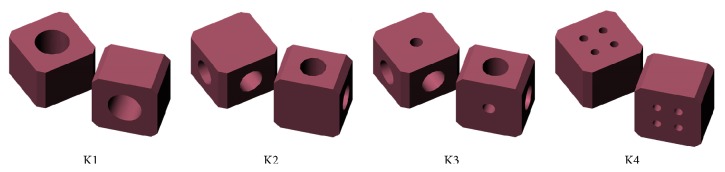
Illustration of the four different cage geometries designed and evaluated with FEM (finite element modelling).

Cages must be able to withstand biomechanical loads and must also leave enough space to place a bone graft for fusion. Four different cage geometries (illustrated in Figure 3) were designed and evaluated with FEM.

Twenty-node solid elements were then used to model the cortical bone, cancellous bone, end plate, and intervertebral disc. Annulus fibrosis was formed by layers that were placed at a 30° angle to one another and that only reacted against distraction. These layers were embedded into ground substance; there were seven layers. A reinforcement element model of the Ansys software was used to define these layers.

The facet joints were modelled as nonlinear contact surfaces:

1. K1 had an 8 mm in diameter cylindrical hall between the upper and lower surfaces.
2. K2 had two 6 mm in diameter cylindrical halls on each side of the cage.
3. K3 had two 6 mm in diameter cylindrical halls on each side, and a 3 mm in diameter cylindrical hall between the upper and lower surfaces.
4. K4 had four 2 mm in diameter cylindrical halls between the upper and lower surfaces.

The base of the L5 vertebra was fixed in every degree of freedom. Loadings were taken from the literature [9]. A 400 N axial compression and 6 Nm bending moment were separately applied, and Von Mises stress distributions were computed for the L4-L5 motion segment.

Biomechanical tests on the processed prototype cage were performed with an Instron testing machine; these tests were simulated with FEM. Two metal platens and the cage were modelled for this purpose. A 5,000 N (5 kN) axial load was applied to the system in FEM.

## 3. Results

### 3.1. Prototype Cage Biomechanical Test

**Figure 4:**
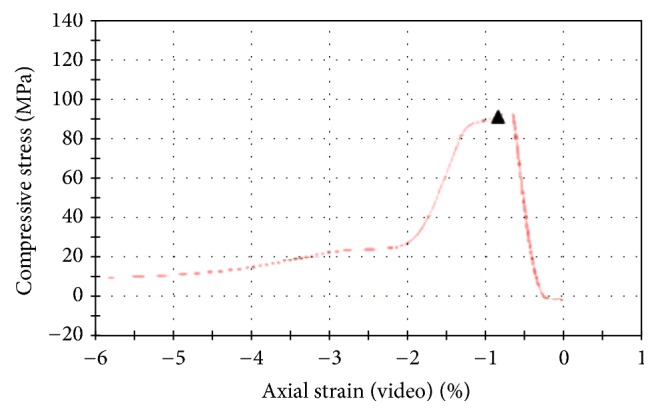
Elastic modulus of the ceramic cage being calculated after the compression test of the prototype cage.

An elastic modulus of the ceramic cage was calculated as 32 GPa after the compression test of the prototype cage; the maximum compressive stress value was 121.16 MPa. Before the failure of the unprocessed cage, the maximum load had been 29.1 kN (Figure 4).

**Figure 5:**
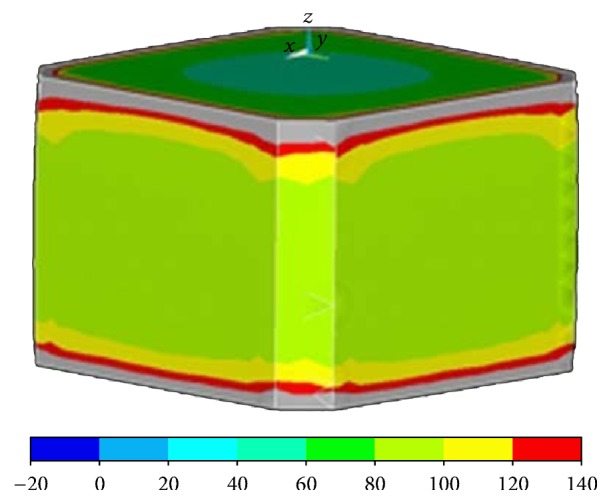
Prototype cage loaded to 25 kN in the FEM. High compression values that exceeded the mechanical capacity were observed on the surfaces within a small area.

The prototype cage was loaded to 25 kN in the FEM; the maximum compression value was 244.0 MPa. When the Von Mises graphics were analyzed, these high values (which exceeded the mechanical capacity) could be observed on the surfaces in a small area. Most of the compression values were between 80 and 100 MPa. These results were similar to those of the real compression test results (Figure 5).

### 3.2. Evaluation of the Cage Geometries with FEM

The maximum stress values and localisations were evaluated; the models included a nonimplanted intact model and four implanted models, using the cage geometries of K1, K2, K3, and K4.

In the intact model, the maximum stress values viewed during flexion, bending, and rotation were found at the intervertebral disc space; during extension, maximum stress value was in the posterior elements. The maximum stress value was 101.4 MPa.

**Figure 6:**
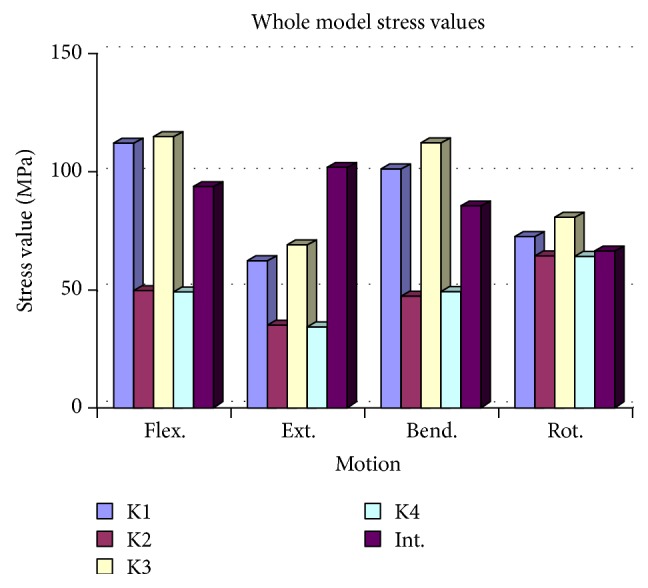
For all modes of loading, maximum stresses in K1 and K3 were observed at the intervertebral space.

The maximum stresses in K1 and K3 were observed at the intervertebral space for all modes of loading; the stress values were higher compared to the intact model for all loadings except extension. The maximum stress values observed in K1 and K3 were 112.1 MPa and 114.6 MPa, respectively (Figure 6).

In the K2 model, the maximum stress values in all modes of loading were observed in the posterior elements and in the posterior instrumentation system. All stress values were found to be lower compared to the intact model. The maximum stress value (63.6 MPa) was observed during rotation (Table 2).

In the K4 model, the maximum stress values were observed on the posterior elements and the posterior instrumentation system, except with bending, where the maximum stress was observed at the intervertebral disc space. All stress values were found to be lower compared to the intact model. The maximum stress value was observed during rotation, at 63.2 MPa (Table 2).

### 3.3. L5 End Plate Compression

**Figure 7:**
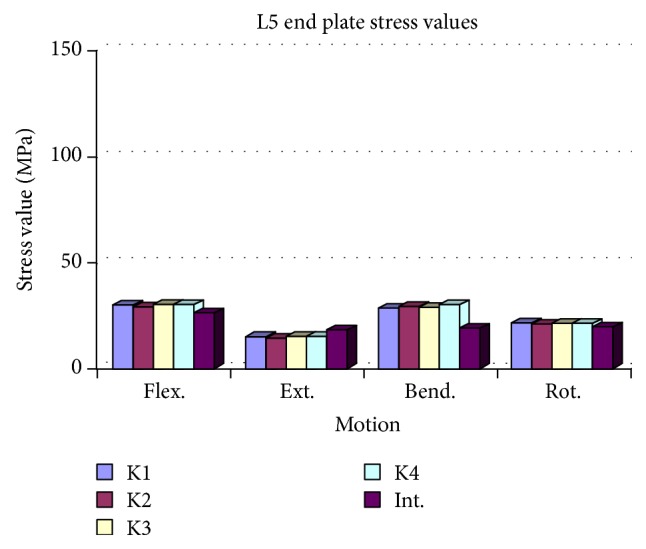
The L5 end plate stress distribution was similar to that of the intact model for flexion, extension, and rotation. The maximum L5 end plate stress value (29.3 MPa) was observed in the K4 model during bending.

The L5 end plate stress distribution was similar to that of the intact model for flexion, extension, and rotation. But for bending, the stress values on the L5 end plate were observed to be significantly higher in all models. The maximum L5 end plate stress value (29.3 MPa) was observed in the K4 model during bending, while the minimum L5 end plate stress value (14.2 MPa) was observed in the K2 model during extension (Figure 7 and Table 3).

### 3.4. Cage Compression

**Figure 8:**
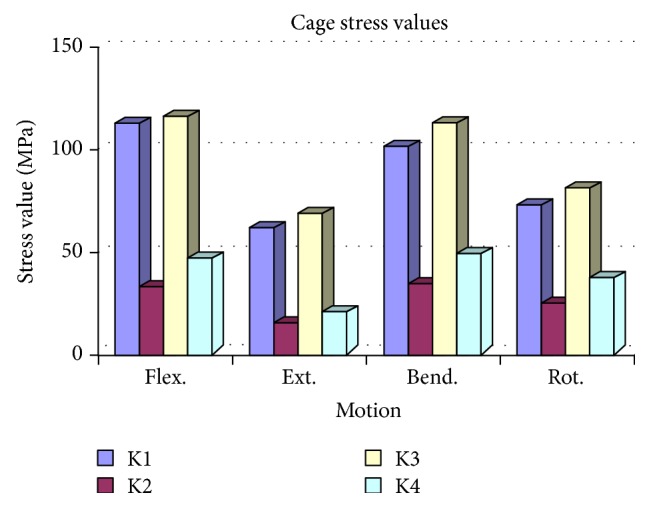
Compared to K2 and K4, maximum stress values of the cages were significantly higher in the K1 and K3 models. The K2 model revealed minimum stress values during all loadings.

In all loadings, the maximum stress values of the cages were significantly higher in the K1 and K3 models compared to K2 and K4. Among all models, the minimum stress values were observed in the K2 model for all loadings. The flexion and bending stress values were found to be higher than extension and rotation in the K2 model (Figure 8 and Table 4).

### 3.5. Biomechanical Test of the Processed Cage

Compression tests were conducted with the processed cage; failure was observed at 4.3 kN for the first sample, 3.9 kN for the second sample, and 5.6 kN for the third sample, for an average of 4.6 kN.

A 5 kN axial compressive loading was applied to the cage, and the Von Mises stresses were analyzed. Stress values were found to be higher at the inner sides and at the corners. The maximum stress at the inner site was 209.4 MPa. The stress values at the processed inner sites were at 80–100 MPa intervals.

## 4. Discussion

At 29.1 kN axial loading, a 121.16 MPa maximum compression stress was experimentally estimated on the prototype cage before failure. An FEM compression test simulation was conducted with the prototype cage, and a maximum axial load of 25 kN was applied. A few small areas exceeded 100 MPa, and the maximum compression stress was found to be 244 MPa but only on the surface of the cage. This high value would appear to be an artefact that was due to modelling assumptions and idealizations at the boundary. The compression stress values of the cage were generally computed to be within an 80–100 MPa interval. According to this analysis (and from our observations from the real compression test), we conclude that failure of the cage will result just above 25 kN, as stress levels at all areas approximate to 29.1 kN. Consistency between the experimental tests that were achieved and the FEM simulations provide confidence that the cage model is indeed realistic.

In an FEM study, Zhong et al. employed topology optimization to design a new cage from an existing titanium cage. They compared the novel cage with the existing cage. After comparison, they designed a new cage that had similar biomechanical performance and more space for bone graft [13].

Our analysis of the K1 and K3 models revealed that stress distribution condensed at the intervertebral space on the cages.

In the K2 model, maximum stress values in all modes of loading were observed on the posterior elements and in the posterior instrumentation system. The maximum stress values detected on the cages were significantly lower than in the intact model. In all compression models and all modes of loading, stress values were detected to be significantly lower than in the K1 and K3 model. These stress levels were within the range of biomechanical performance that would be acceptable for the A/W bioceramic composite. The K2 model demonstrated that the posterior elements and the posterior instruments were effective in load sharing and decreased the loads on the cages; thus, increased loading at the anterior compartment of the spine could enhance fusion. Although the stress values of the K2 and K4 models were computed to be similar, the values of the K4 model were minimally higher compared to the K2 model.

In addition to adequate biomechanical performance, an ideal cage design should have enough space for bone grafting. In the current study, the best biomechanical performance was computed in the K2 model. It should also be noted that there was enough space for a bone graft in this model. Similar to K3, by drilling an extra hole in the upper surface of the K2 model, the space for a bone graft was widened. Hence, this alteration significantly led to increased stress.

Similarly, in the K1 model, the hole in the upper surface significantly increased the stress values. In order to avoid this problem, the dimension of the hole might be decreased, but this could lead to narrowing of the space for the graft.

The K4 model was found to be biomechanically inferior to the K2 model; the space available inside the K4 model was also narrower than in K2.

In an FEM study, Vadapalli et al. compared titanium and PEEK cages and found that the stress values of vertebrae end plates for titanium cages were 2.5 times higher than for PEEK cages; they concluded that maximum stress values (48 MPa for the titanium cage and 20 MPa for the PEEK cage) were observed during a bending load [11]. In the current bioceramic composite cage study, the maximum end plate stress value was detected (at 28.9 MPa) under the same bending load. This value was minimally higher than the value of the PEEK cage but was significantly lower than that of the titanium cage. Therefore the A/W bioceramic composite cage shows similar risk to the PEEK cage (and significantly lower risk than the titanium cage) for end plate fracture formation.

Interbody cages are generally used with posterior instrumentation systems; in clinical procedures, posterior instrument failures are observed more than cage failures [14]. In another FEM study, Zhong et al. used a titanium cage and subjected the lumbar spine to 10 Nm flexion, extension, torsion, and lateral bending moment with 150 N axial preload. At these loading conditions, the stress values on the posterior instruments in flexion, extension, bending, and rotation were 73.4 MPa, 61 MPa, 77.3 MPa, and 96.1 MPa, respectively. In the current study, the K2 model's axial preload was 400 N, and the moment was 6 Nm for each mode of loading. The stress values on the posterior instruments on flexion, extension, bending, and rotation were 48.8 MPa, 33.9 MPa, 46 MPa, and 63.6 MPa, respectively. Compared to the current study, the loading conditions were different in Zhong et al.'s study in terms of higher moment and lower axial preload. Although the loading conditions were different in the aforementioned two studies, the stress values that were compared in the posterior elements turned out to be lower in the K2 model. These data suggest that the A/W bioceramic composite cage can carry enough load at the anterior part of the spine and can support stabilization well.

After the compression of the three processed cages, the mean failure value was 4.6 kN in the axial loading. We believe that the different biomechanical performances may have been due to microfractures that had occurred during the cage process. These differences will not be observed if these cages are fabricated via moulding.

A K2 cage compression test was also simulated with FEM. In this test, the axial load applied was 5 kN, which was close to the mean value (4.6 kN) of the real test. The stress values were in the range of 80–100 MPa at the centre. We thought that, by increasing the axial load, the stress would have increased and we would have observed the failure of the cage. A comparison between the real test and the FEM test found that they were concordant. Although the FE model that was used in the current study utilized literature-derived material properties, the main limitation of our study is that experimental validation of the FEM is lacking. Another limitation of our study is not to perform cycling loading and impact tests to define the material properties of A/W bioceramic. Our aim was to produce a prototype A/W bioceramic cage for spinal fusion. For this purpose, we tested A/W bioceramic cage with FEM. However, in order to define the material properties and validate the FEM, we only performed biomechanical compression tests. We would conduct advanced biomechanical analyses including cyclic loading and impact tests in the future studies.

In conclusion, the FEM simulation has the advantage of demonstrating the relationship and mechanism between implant and related spinal segments. The A/W bioceramic composite is a bioactive and compatible material that can effectively be used for interbody fusion. We must also keep in mind that the design of the cage plays a crucial role (in addition to its material properties) for achieving stable fusion. Although the K2 bioceramic composite cage model was found to have favorable biomechanical performance, animal studies must be conducted to elucidate the optimal and final design for this cage.

## Figures and Tables

**Table 1:** Material properties of the tissues within the spinal column [11]. ALL: anterior longitudinal ligament; PLL: posterior longitudinal ligament; TL: transverse ligament; LF: ligamentum flavum; ISL: interspinous ligament; SSL: supraspinous ligament; CL: capsular ligament.

Material	Elastic modulus	Poisson coefficient	Cross-sectional area
Cortical bone	12000	0.3	—
Cancellous bone	100	0.2	—
Posterior elements	3500	0.25	—
Intervertebral disc			
Nucleus	1	0.499	
Ground substance	4.2	0.45	
Annulus fibers	450	—	0.76
End plate	24	0.4	
Ligaments			
ALL	20	—	63,7
PLL	20	—	20
TL	58.7	—	3.6
LF	19.5	—	40
ISL	11.6	—	40
SSL	15	—	30
KL	32.9	—	60
Titanium	110000	0.28	—

**Table 2:** Maximum stress values of the whole construct and their localisations under different loads. The maximum stress values of the whole construct by using K2 and K4 designs were significantly better (*p* < 0.05). I: intact model.

	Flexion		Extension		Bending		Rotation	
	Max. stress value	Max. stress point	Max. stress value	Max. stress point	Max. stress value	Max. stress point	Max. stress value	Max. stress point
I	93.5	Disc space	101.4	Posterior elements	85.2	Disc space	66.0	Disc space
K1	112.1	Disc space	61.6	Disc space	100.8	Disc space	72.3	Disc space
K2	48.8	Posterior elements	33.9	Posterior elements	46.0	Posterior elements	63.6	Posterior elements
K3	114.6	Disc space	68.4	Disc space	112.1	Disc space	80.6	Disc space
K4	48.7	Posterior elements	33.6	Posterior elements	48.6	Disc space	63.2	Posterior elements

**Table 3:** Maximum stress values in the L5 end plate and their localisations under different loads. There were no significant differences between cage designs (*p* > 0.05). I: intact model.

	Flexion		Extension		Bending		Rotation	
	Max. stress value	Max. stress point	Max. stress value	Max. stress point	Max. stress value	Max. stress point	Max. stress value	Max. stress point
I	25.9	Anterior	17.1	Posterior	17.7	Anterolateral	18.4	Anterior
K1	28.9	Anterior	14.7	Anterior	27.9	Anterolateral	20.5	Anterior
K2	28.0	Anterior	14.2	Anterior	28.9	Anterolateral	20.0	Anterior
K3	29.0	Anterior	14.8	Anterior	27.9	Anterolateral	20.5	Anterior
K4	28.9	Anterior	14.6	Anterior	29.3	Anterolateral	20.5	Anterior

**Table 4:** Maximum stress values of the different cage designs. The stress values were significantly lower in the K2 design (*p* < 0.05).

	Flexion		Extension		Bending		Rotation	
	Max. stress value	Max. stress point	Max. stress value	Max. stress point	Max. stress value	Max. stress point	Max. stress value	Max. stress point
K1	112.1	Anterior	61.6	Posterior	100.8	Anterolateral	72.3	Anterior
K2	32.6	Anterior	15.3	Posterior	33.7	Anterolateral	24.3	Anterior
K3	114.6	Anterior	68.4	Posterior	112.1	Anterolateral	80.6	Anterior
K4	46.3	Anterior	20.2	Anterior	48.6	Anterolateral	36.8	Anterior
